# Data-driven discovery of chemotactic migration of bacteria via coordinate-invariant machine learning

**DOI:** 10.1186/s12859-024-05929-w

**Published:** 2024-10-24

**Authors:** Yorgos M. Psarellis, Seungjoon Lee, Tapomoy Bhattacharjee, Sujit S. Datta, Juan M. Bello-Rivas, Ioannis G. Kevrekidis

**Affiliations:** 1 Department of Chemical and Biomolecular Engineering, Johns Hopkins University, Baltimore, MD USA; 2 Department of Mathematics and Statistics, California State University, Long Beach, Long Beach, CA USA; 3 Andlinger Center for Energy and the Environment, Princeton University, Princeton, NJ USA; 4 Department of Chemical and Biological Engineering, Princeton University, Princeton, NJ USA; 5 Department of Applied Mathematics and Statistics, Johns Hopkins University, Baltimore, MD USA; 6 Department of Medicine, Johns Hopkins University, Baltimore, MD USA

**Keywords:** Chemotaxis, Partial differential equations, Machine learning, Neural letworks

## Abstract

**Background:**

*E. coli* chemotactic motion in the presence of a chemonutrient field can be studied using wet laboratory experiments or macroscale-level partial differential equations (PDEs) (among others). Bridging experimental measurements and chemotactic Partial Differential Equations requires knowledge of the evolution of all underlying fields, initial and boundary conditions, and often necessitates strong assumptions. In this work, we propose machine learning approaches, along with ideas from the Whitney and Takens embedding theorems, to circumvent these challenges.

**Results:**

Machine learning approaches for identifying underlying PDEs were (a) validated through the use of simulation data from established continuum models and (b) used to infer chemotactic PDEs from experimental data. Such data-driven models were surrogates either for the entire chemotactic PDE right-hand-side (black box models), or, in a more targeted fashion, just for the chemotactic term (gray box models). Furthermore, it was demonstrated that a short history of bacterial density may compensate for the missing measurements of the field of chemonutrient concentration. In fact, given reasonable conditions, such a short history of bacterial density measurements could even be used to *infer* chemonutrient concentration.

**Conclusion:**

Data-driven PDEs are an important modeling tool when studying Chemotaxis at the macroscale, as they can learn bacterial motility from various data sources, fidelities (here, computational models, experiments) or coordinate systems. The resulting data-driven PDEs can then be simulated to reproduce/predict computational or experimental bacterial density profile data independent of the coordinate system, approximate meaningful parameters or functional terms, and even possibly estimate the underlying (unmeasured) chemonutrient field evolution.

**Supplementary Information:**

The online version contains supplementary material available at 10.1186/s12859-024-05929-w.

## Background

We study models of the phenomenon of chemotactic migration of bacteria, i.e. their ability to direct multicellular motion along chemical gradients. This phenomenon is central to environmental, medical and agricultural processes [1].

Chemotaxis can be studied at several (complementary) levels [2]: extensive fundamental research focuses on understanding the cellular mechanisms behind sensing chemoattractants/chemorepellents and how they induce a motility bias [3–5]. Another approach is to understand and simulate chemotaxis in the context of stochastic processes and Monte Carlo methods [6–9]. It is also possible, at appropriate limits, to employ macroscopic PDEs to simulate the spatiotemporal evolution of bacterial density profiles in the presence of a chemoattractant/chemorepellent field [10]. In the latter approach, the Keller-Segel model has been historically successful [11]:

1 $$\frac{{\partial b}}{{\partial t}} = \nabla \cdot \left( {D\nabla b - b\chi (S)\nabla S} \right),{\text{ }}$$

where *b*(*x*, *t*) denotes the bacterial density, *D* the diffusion coefficient and *S* is the spatial field of the chemoattractant/chemorepellent (here considered constant in time). This model explicitly describes cell motility through two terms: a diffusion term (usually isotropic) and a chemotactic term, which encapsulates the response of the bacteria in the presence of a chemoattractant/chemorepellent field. This response includes signal transduction dynamics and properties of cellular chemoreceptors. In this term, the function $\chi : \mathbb {R} \rightarrow \mathbb {R}$ can be tuned for different kinds of chemotactically relevant substances and their spatial profile. Most importantly, the sign of $\chi$ distinguishes chemoattractants vs. chemorepellents. In general, the dynamics of *S* are described by a second PDE (for the field *S*(*x*, *t*)) coupled with the one above (see Eq. 10). In this work, we will deal only with chemoattractants (and specifically chemonutrients).

Despite the generality and applicability of the Keller-Segel model, a quantitative closed-form formula for the chemotactic term is very difficult, or even impossible, to obtain. For example, to model the chemotactic motion of *Escherichia coli (E. coli)* in heterogeneous porous media, Bhattacharjee et al., have used an extension of the Keller-Segel model [1] (see Eq. 10). This model includes logarithmic sensing (Weber-Fechner Law) an experimentally validated phenomenon [4, 12, 13] and has been used in the past to understand chemotaxis experiments [14, 15]. In that model, bacteria bias their motion towards the chemonutrient which they can consume (see second PDE in Eq. 10). In that process, and for initial conditions corresponding to the experimental data presented here, they exhibit a macroscopic coherent propagating “bacterial wave”.

In this work, we demonstrate a toolbox of machine learning methodologies that help learn different forms of *the law* of macroscopic chemotactic partial differential equations, either from simulations or from experimental data. These methods essentially help construct macroscopic surrogate models either (a) for the entire right-hand-side of the PDEs (black box) or (b) for some of the right-hand-side terms that are analytically unavailable/intractable while the remaining are known (gray box model). Even though the functional form of the PDE right-hand-side cannot be analytically recovered, it is efficiently approximated by machine learning algorithms guaranteeing universal approximation.

Understanding and predicting the behavior of such a complex system is always a challenge. When the single-agent dynamics are known (possibly from first principles), the system can be studied and simulated at the microscale. Macroscale behavior naturally arises from simulating sufficiently large agent ensembles. It is sometimes possible to derive macroscale partial differential equations from the dynamics of the individual agents [9, 16]. Such PDE-level descriptions are particularly attractive, as we are usually interested in the evolution of only a few, important macroscopic variables, rather than of each individual “microscopic” agent (i.e. each individual bacterium). Importantly, one also needs to know which (and how many) macroscopic variables/observables are sufficient to usefully construct a closed macroscopic evolution equation (e.g. [17]).

For systems of great complexity, an accurate macroscopic PDE may be out of reach. One could only gather (full or partial) information from experiments and/or fine-scale, individual based/stochastic simulations. This calls for a data-driven approach to “discover" a macroscopic law for a coarse PDE, solely from spatiotemporal data (experimental or computational movies). Such a data-driven PDE can then be exploited towards the following purposes:

1. Predicting the time evolution starting from different initial conditions or in out-of-sample spatiotemporal domains. This is particularly attractive when it is not easy to probe the system and extract such profiles “on demand” from experiments or simulations. On the contrary, such data-driven models can provide predictions without the need to recalibrate/retrain for different scenarios, owing to the generalizability of the (learned) PDE laws. Note that, as in any data-driven modeling approach, generalizability strongly depends on the training data selection (see [9] for a discussion and example).
2. Reconstructing the full behavior of the system even when only partial information is at hand (i.e. when we do not have data for all important macroscopic variables). This is particularly interesting in the case where chemonutrient concentrations are difficult to observe due to experimental limitations [18].
3. If a qualitatively correct but quantitatively inaccurate macroscopic model happens to be available, a quantitative data-driven model can help probe and even understand different components of the system’s behavior [9]. This can be a way to shed light on the fundamental physical laws of the studied system (explainability).

Our work falls in the general category of nonlinear system identification using data-driven, machine-learning-assisted surrogate models. Neural Networks have repeatedly demonstrated successes in learning nonlinear Ordinary [19] or Partial Differential Equations [17, 20]. More recently, with the increased accessibility of powerful computational hardware and the computational efficiency of machine learning algorithms, nonlinear system identification has attracted a lot of attention [21–24] and has motivated the design of novel approaches and architectures. Notable among these approaches are Neural ODEs [25] and Convolutional Neural Networks [26, 27], sparse identification [28] and effective dynamics identification [29, 30].

It is worth making a distinction between our approach and physics-informed Neural Networks (PINNS, [23]). Even though our approach allows for the incorporation of some physics knowledge (i.e. part of the PDE might be known, as in our gray boxes) our goal remains the *discovery of the law* of the PDE, or terms thereof. On the contrary, PINNS aim at approximating *the solution of the PDE* ***knowing the law***. A major difference, is that in PINNs the solution is obtained for a specific configuration of spatiotemporal domain, initial and boundary conditions. In principle, learning a (local, PDE) law instead, allows for data-driven modeling in *any* such configuration. A possible byproduct of PINNS could be parameter estimation [23, 31] but this still differs significantly from system identification, as we do.

## Models and results

We constructed data-driven models for two different data sources (which, however correspond to the same general chemotaxis scenario):

(i) PDE simulation data: The Chemotactic PDE described and used in [1] was simulated and data were collected for both bacterial density and nutrient concentration fields. Details about this PDE and its simulation can be found in Materials and Methods.
(ii) Real-world data from chemotaxis experiments were used. These experiments were performed by Bhattacharjee et al. and described in [1].

The results are presented separately for each of these two categories.

### Models for simulation data

**Fig. 1:**
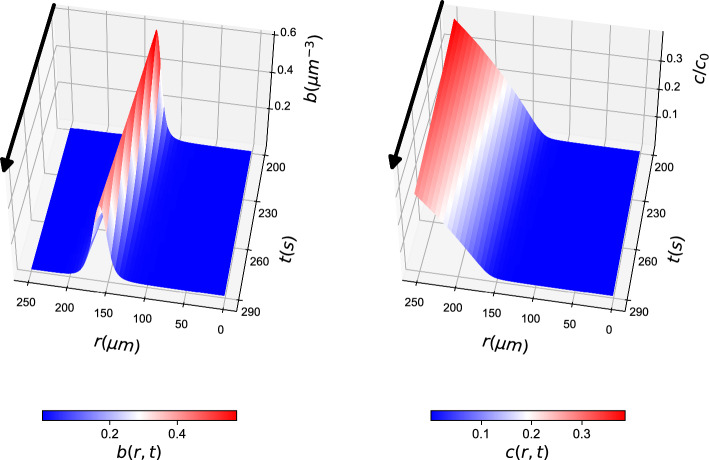
PDE simulations representing the ground truth of the simulations: (left) *b*(*r*, *t*) field and (right) *c*(*r*, *t*) field. For clarity, arrows are added to denote the direction of time

PDE simulations included the integration of two coupled PDE fields, one describing the bacterial density *b* and one describing the nutrient density *c* (System 10). The PDE solution can be seen in Fig. 1.

**Table 1:** Listing of all data-driven models explored in the manuscript.

Model	Surrogate function	Known fields	Known RHSs	Output	Algorithm
Black box for 2 PDEs	$f_{GP}, h_{GP}$	*b*(*r*, *t*), *c*(*r*, *t*)	–	$b_t, c_t$	GPR
	$f_{NN}, h_{NN}$	*b*(*r*, *t*), *c*(*r*, *t*)	–	$b_t, c_t$	ANN
Black box for 1 PDE	$f_{GP}$	*b*(*r*, *t*), *c*(*r*, *t*)	$c_t$	$b_t$	GPR
	$f_{NN}$	*b*(*r*, *t*), *c*(*r*, *t*)	$c_t$	$b_t$	ANN
Black box, delays	$f^{partial}_{GP}$	*b*(*r*, *t*), history	–	$b(r, t+\Delta t)$	GPR
	$f^{partial}_{NN}$	*b*(*r*, *t*), history	–	$b(r, t+\Delta t)$	ANN
Gray box	$g_{GP}$	*b*(*r*, *t*), *c*(*r*, *t*)	$c_t$	$b_t-D_b\Delta b$	GPR
	$g_{NN}$	*b*(*r*, *t*), *c*(*r*, *t*)	$c_t$	$b_t-D_b\Delta b$	ANN
Gray box, delays	$g^{partial}_{GP}$	*b*(*r*, *t*), history	–	$b(r, t+\Delta t)-D_b\Delta b(r,t)$	GPR
	$g^{partial}_{NN}$	*b*(*r*, *t*), history	–	$b(r, t+\Delta t)-D_b\Delta b(r,t)$	ANN

*f*, *h* denote surrogate functions for the entire RHS of the $b-$ and $c-$PDE respectively, while *g* denotes a surrogate for the chemotactic term. Subscripts “GPR” and “NN” denote Gaussian Process Regression and Artificial Neural Network respectively. $\Delta t$ denotes the time delay used in all models with partial information. Only the models in rows indexed 2,3,4,6,8  will be presented in detail in the following sections; the rest are included in the Supplementary Information

**Table 2:** Summary of all data-driven models for learning the nutrient field (denoted as *c*(*x*, *t*)). Only the second  model will be presented in detail in this section. The other model is included in the Supplementary Information

Surrogate function	Known fields	Known RHSs	Output	Algorithm
$C_{GP}$	*b*(*r*, *t*), history	–	*c*(*r*, *t*)	GPR
$C_{NN}$	*b*(*r*, *t*), history	–	*c*(*r*, *t*)	ANN

To learn from simulation data, we considered a variety of machine-learning-enabled data-driven models; they are listed in Tables1 and 2; the relevant notation is summarized in the Table captions. In the text that follows, a representative selection (see the captions in Tables 1, 2) will be described in more detail; the remaining ones are relegated to the Supplementary Information.

#### Black box data-driven models

Consider a system described by *d* macroscopic scalar variable fields ($u^{(1)},..., u^{(d)}$). Assuming a one-dimensional domain along the vector $\hat{x}$ (for an example in cylindrical coordinates see Fig. 12) discretized through *m* points in space (*x*) and *n* points in time (*t*), we are given $m\cdot n$ data points in $\mathbb {R}^{d}$. Using interpolation/numerical differentiation, we can estimate the temporal derivatives $u^{(1)}_t,..., u^{(d)}_t$, as well as various order derivatives in space (first, $u^{(1)}_x,..., u^{(d)}_x$, second $u^{(1)}_{xx},..., u^{(d)}_{xx}$, etc). We assume that we know *a priori* the largest order of relevant spatial derivatives (here, two) [32], the coordinate system, and the boundary conditions (here, zero flux). We also assume that the spatiotemporal discretization satisfies the necessary criteria for a numerically converged PDE solution. Given these derivatives, we can compute all relevant local operators, such as: $\mathbf {\nabla u^{(i)}}, \Delta u^{(i)}, i \in \{1,..., d\}$.

Our objective is to learn functions $f_i: \mathbb {R}^{3d \cdot m} \rightarrow \mathbb {R}^m, i \in \{1,..., d\}$ such that:

2 $$u_t^{(i)} = {f_i}({u^{(1)}},...,{u^{(d)}},\nabla {u^{(1)}} \cdot \hat x,...,\nabla {u^{(d)}} \cdot \hat x,\Delta {u^{(1)}},...,\Delta {u^{(d)}})$$

This is a **black box expression** for the time derivative of a macroscopic field expressed as a function of the relevant lower order *coordinate-independent* local spatial operators, operating on the fields. The coordinate-independent nature of the input features, allows the use of such data-driven laws across different coordinate systems, as well as fusion of data from different coordinate systems in the training. This property, we believe, is of great importance as it results in versatile, generalizable models. After training (after successfully learning this function based on data) integrating this model numerically can reproduce spatiotemporal profiles in the training set, and even hopefully predict them beyond the training set. The arguments of $f_i$ will be the features (or input vectors) and $u^{(i)}_t$ will be the target (or output vector) of our data-driven model. Note that, usually, not all features are informative in the learning of $f_i$ (in other words, only some orders of the spatial derivatives appear in the PDE right-hand-side). Also, note that not all macroscopic variables $u^{(i)}$ are always necessary for learning $f_j, j \ne i$. In the spirit of the Whitney and Takens embedding theorems [33, 34], short histories of some of the relevant variable profiles may “substitute” for missing observations/measurements of other relevant variables.

#### Coordinate invariant learning

In Cartesian coordinates the operators used as inputs to learn right-hand sides (or, here, chemotactic terms) are simply related to the spatial derivatives; but in curvilinear coordinates, or when the evolution occurs on curved manifolds, the relation between spatial derivatives and local operators needs more care. We consider physical Euclidean space $\mathbb {R}^3$ (regarded as a Riemannian manifold with Euclidean metric expressed as $g = (\textrm{d}x)^2 + (\textrm{d}y)^2 + (\textrm{d}z)^2$ in Cartesian coordinates (*x*, *y*, *z*)). The gradient, ${{\,\textrm{grad}\,}}f$, of a smooth function $f :\mathbb {R}^3 \rightarrow \mathbb {R}$ is a vector pointing in the direction at which *f* grows at its maximal rate and whose length is said maximal rate —note that this definition is independent of the system of coordinates on $\mathbb {R}^3$. The phase flow of a smooth vector field $v :\mathbb {R}^3 \rightarrow \mathbb {R}^3$ can be regarded as the motion of a fluid in space. The divergence of *v*, denoted by $\mathrm{div}\hspace{0.1cm}v$, is the outflow of fluid per unit volume at a given point. Again, the definition is coordinate frame independent; the expression $\mathrm{div}\hspace{0.1cm} v = \frac{\partial v_1}{\partial x} + \frac{\partial v_2}{\partial y} + \frac{\partial v_3}{\partial z}$ is valid in Cartesian coordinates. The Laplacian is the composition of the gradient followed by the divergence (in other words, $\Delta = \mathrm{div} {{\,\textrm{grad}\,}}$), again independently of the choice of coordinates.

Note that in our specific case of **cylindrical coordinates** with only the component along the radial dimension (with unit vector denoted $\hat r$) being important due to domain-specific symmetries, the right-hand-side of a PDE will explicitly depend on the local radius as well. In the above formulation, this is incorporated in the Laplacian term $\Delta u^{(i)} = \frac{1}{r} \frac{\partial }{\partial r} (r \frac{\partial u^{(i)}}{\partial r} )$. For the construction of all relevant differential operators in any coordinate system, it is possible to use ideas and tools from Exterior Calculus (see Supplementary Information). In the Supplementary Information we also include some case studies that demonstrate the coordinate-invariance of our proposed algorithmic framework.

*Black box learning of both PDEs with an ANN (from known fields* *b*(*r*, *t*), *c*(*r*, *t*))

3 $$\left[ {\begin{array}{*{20}{c}} {{b_t}} \\ {{c_t}} \end{array}} \right] = \left[ {\begin{array}{*{20}{c}} f \\ h \end{array}} \right] = {F_{NN}}(b,\nabla b \cdot \hat r,\Delta b,c,\nabla c \cdot \hat r,\Delta c)$$

**Fig. 2:**
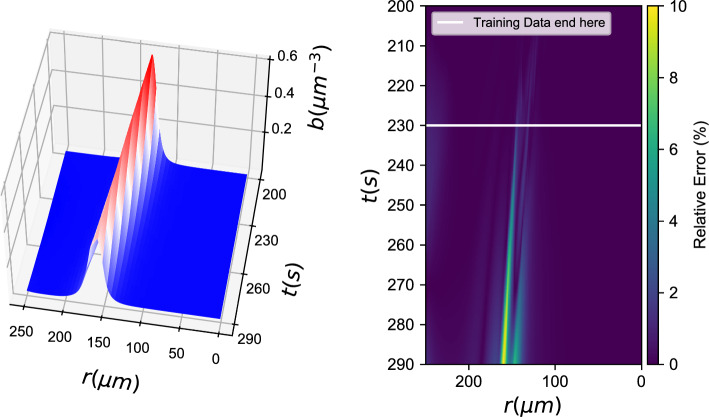
Black box learning of both PDEs with an Artificial Neural Network: (left) Integration results for the **first** data-driven PDE (for *b*(*r*, *t*)) and (right) relative error (%)

**Fig. 3:**
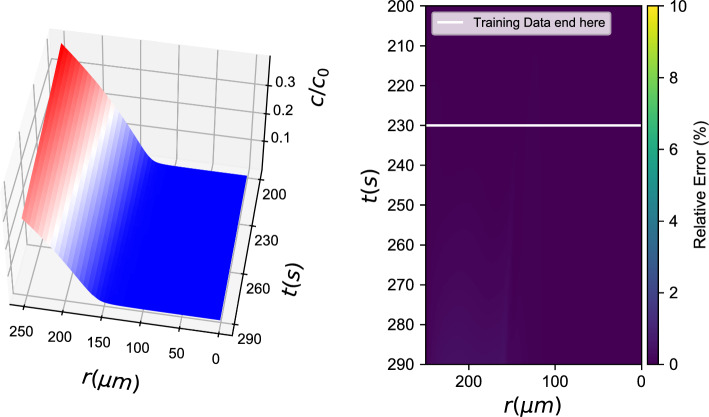
Black box learning of both PDEs with an Artificial Neural Network: (left) Integration results for the **second** data-driven PDE (for *c*(*r*, *t*)) and (right) relative error (%)

With training data from both *b*(*r*, *t*) and *c*(*r*, *t*) fields, we learned a neural network of the form described in Eq. 3. Figures 2, 3 show how the data-driven PDEs were able to learn the laws of time evolution of both *b*(*r*, *t*) and *c*(*r*, *t*). The data-driven models were used to reproduce the training data and could successfully extrapolate as far as we attempted (here, up to $t=290\textrm{s}$).

*Black box learning of* $b_t$ *with ANN* - $c_t$ *known (with fields* *b*(*r*, *t*), *c*(*r*, *t*) *known)*

4 $${b_t} = {f_{NN}}(b,\nabla b \cdot \hat r,\Delta b,c,\nabla c \cdot \hat r,\Delta c)$$

**Fig. 4:**
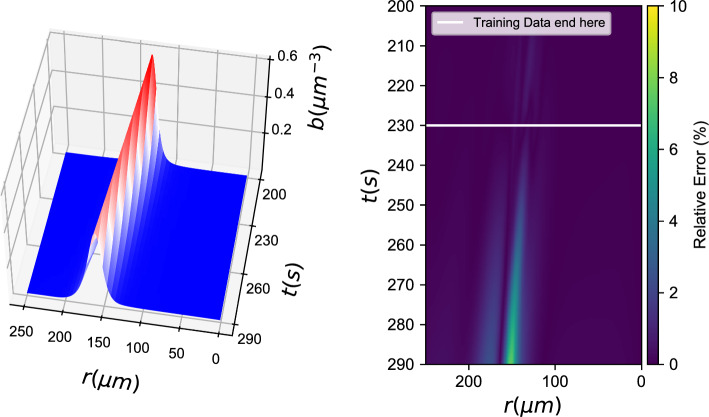
Black box learning with a Neural Network: (left) Integration results for the data-driven PDE and (right) % relative error

**Fig. 5:**
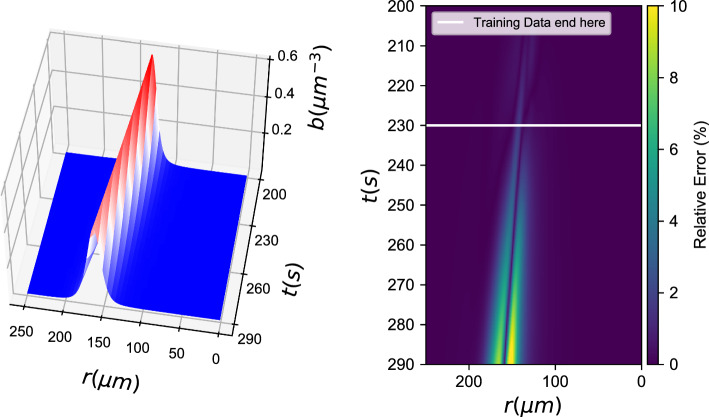
Black box learning with Gaussian Process Regression: (left) Integration results for the data-driven PDE and (right) % relative error

Figure 4 showcases learning of *one of the two* data-driven PDEs when the second PDE is known. Here, the chemonutrient (*c*(*r*, *t*)) PDE is assumed known, and the bacterial density evolution PDE is learned with a neural network, similar to Eq. 3. The trained ANN is able to approximate the ground truth PDE right-hand-side in the proximity of the training dataset. To assess the ANN’s approximation ability, one can look at the norm of the difference between ground truth (“functional”) and ANN approximated outputs for the same inputs: $||b_t^{functional} -b_t^{ANN}||_2^2$. For a data cloud close to the training data ($\pm 10\%$ in each direction) this norm represents an average relative error in the order of 2%. For comparison purposes, we also trained a Gaussian Process Regression algorithm for the same task (see. Fig. 5).

*Black box ANN learning of a single field evolution equation, with only partial information (only the field* *b*(*r*, *t*) *is observed)*

After the initial success of the previous section, we decided to attempt a computational experiment, based on the spirit of the Takens embedding for finite-dimensional dynamical systems [33–38].

The idea here is that, if only observations of a few (or even only one) variables involved are available, one can use history of these observations (“time-delay” measurements) to create a useful latent space in which to embed the dynamics -and in which, therefore, to learn a surrogate model with less variables, but involving also history of these variables [39, 40]. There are important assumptions here: finite (even low) dimensionality of the long-term dynamics, something easier to contemplate for ODEs, but possible for PDEs with attracting, low dimensional, (possibly inertial) manifolds for their long-term dynamics. There is also the assumption that the variable whose values and history we measure is a *generic observer* for the dynamics on this manifold.

One can always claim that, if a 100-point finite difference discretization of our problem is deemed accurate (so, for two fields, 200 degrees of freedom), then the current discretized observation of one of the two fields (100 measurements) plus three delayed observations of it ($3 \times 100$) plus possibly one more measurement give us enough variables for a useful latent space in which to learn a surrogate model. Here we attempted to do it with much less: at each discretization point we attempted keeping the current *b*(*r*, *t*) field measurement and its spatial derivatives, and added only some history (the values and spatial derivatives at the previous moment in time). The equation below is written in discrete time form (notice the dependence of the field at the next time step from two previous time steps); it can be thought of as a discretization of a *second order in time* partial differential equation for the *b*(*r*, *t*) field, based on its current value and its recent history.

5 $$b({t_{k + 1}}) = b({t_k}) + \Delta tf_{NN}^{partial}(b({t_k}),(\nabla b \cdot \hat r)({t_k}),(\Delta b)({t_k}),;\quad b({t_{k - 1}}),(\nabla b \cdot \hat r)({t_{k - 1}}),(\Delta b)({t_{k - 1}})),$$

with $\Delta t = t_{k+1} - t_k$, for any time point $t_k, k \geqslant 1$.

**Fig. 6:**
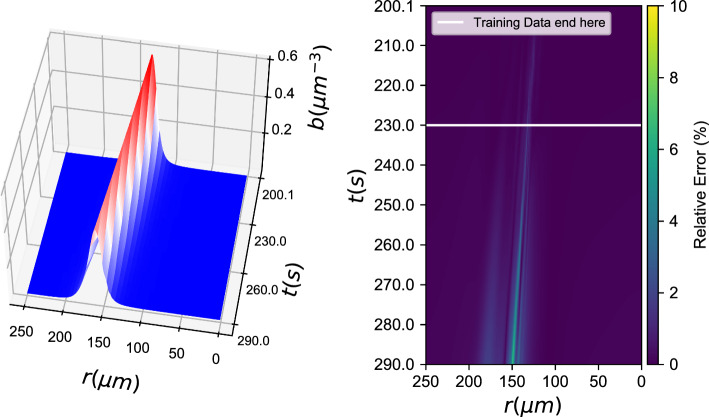
Black box partial-information learning with a Neural Network: (left) Integration results for the data-driven PDE and (right) % relative error

Figure 6 demonstrates learning a data-driven (discrete in time here) evolution equation for the bacterial density *b*(*r*, *t*) when only data for *b*(*r*, *t*) are at hand (partial information). Even though we knew the existence of another, physically coupled field, we assumed we cannot sample from it, so we replaced its effect on the *b*(*r*, *t*) field through a functional dependence on the history of *b*(*r*, *t*). Simulation of the resulting model was successful in reproducing the training data and extrapolating beyond them in time.

#### Gray box data-driven models

A similar approach can be implemented when we have knowledge of a term/ some of the terms **but not of the rest of the terms** of the right-hand side. In the specific context of chemotaxis, we are interested in learning just the chemotactic term, i.e. functions $g_i: \mathbb {R}^{3d\cdot m} \rightarrow \mathbb {R}^m, i \in \{1,..., d\}$ such that:

6 $$u_t^{(i)} - {D^{(i)}}\Delta {u^{(i)}} = {g_i}({u^{(1)}},...,{u^{(d)}},\nabla {u^{(1)}} \cdot \hat x,...,\nabla {u^{(d)}} \cdot \hat x,\Delta {u^{(1)}},...,\Delta {u^{(d)}}),$$

where $D^{(i)}$ is an *a priori* known diffusivity. This is now a **gray box model** for the macroscopic PDE, and is particularly useful in cases where an (effective) diffusion coefficient is easy to determine, possibly from a separate set of experiments or simulations [9]. Again, as for black box models, gray boxes can also be formulated in the case of partial information, i.e. when not all fields $u^{(i)}$ are known, by leveraging history information of the known variables. Note that this framework (but also the black box one) can be adjusted to account for parametric dependence, which enables further downstream objectives, such as bifurcation studies or parameter estimation. See Eq. 8 for a demonstration of a gray box model used to identify the upper and lower characteristic bounds of logarithmic sensing [1].

*Gray box learning with ANN* - $c_t$ *known (with fields* *b*(*r*, *t*), *c*(*r*, *t*) *known)*

7 $${b_t} - {D_b}\Delta b = {g_{NN}}(b,\nabla b \cdot \hat r,\Delta b,c,\nabla c \cdot \hat r,\Delta c)$$

**Fig. 7:**
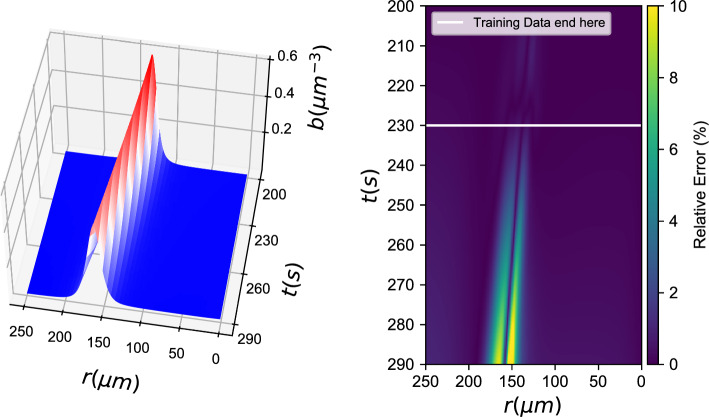
Gray box learning with a Neural Network: (left) Integration results for the data-driven PDE and (right) % relative error

Figure 7 shows the performance of gray box models, where only the chemotactic term of the bacteria density PDE was considered unknown. For this gray box model, the effective diffusion coefficient for the bacterial density was considered known. In principle, one could also hardwire the knowledge *of the functional form of this term* in the loss of the neural network, and thus obtain an estimate of this diffusivity in addition to learning the chemotactic term in the PDE.

As mentioned before, the gray box framework can be adjusted to account for parametric dependence:

8 $${b_t} - {D_b}\Delta b = {g_{NN*}}(b,\nabla b \cdot \hat r,\Delta b,c,\nabla c \cdot \hat r,\Delta c,p),$$

where $\mathbf {p=(c_+, c_-)}$ here describes the upper and lower characteristic bounds of logarithmic sensing. After generating an appropriate dataset and training an ANN (see Methods for details), it was possible to recover the parameter vector ${\textbf{p}}$ used in our simulation, by minimizing:

9 $$\mathcal{L}(p) = ||{b_t} - {D_b}\Delta b - {g_{NN*}}(b,\nabla b \cdot \hat r,\Delta b,c,\nabla c \cdot \hat r,\Delta c,p)||_2^2.$$

Specifically, the ground truth parameter vector was ${\textbf{p}}=(30,1) \mu M$ while the recovered ${\textbf{p}}=(31.63, 1.48)\mu M$ (average value).

#### Estimating the chemonutrient field–computational data.

Following up the above success in using Takens’ embeddings (and more generally, Whitney embeddings) [33, 34] for low-dimensional long-term dynamics, we attempted to estimate (i.e. create a nonlinear observer - a “soft sensor” of) the chemonutrient field from local measurements of the bacterial fields and its history [41, 42]. More specifically, we attempted to train a neural network to learn (in a data driven manner) $c(r_i,t_k)$ as a function of some local space time information:

8a $$c({r_i},{t_k}) = {C_{NN}}(b({r_i},{t_k}),(\nabla b \cdot \hat r)({r_i},{t_k}),(\Delta b)({r_i},{t_k}),b({r_i},{t_{k - 1}}),(\nabla b \cdot \hat r)({r_i},{t_{k - 1}}),(\Delta b)({r_i},{t_{k - 1}})),$$

for any discretization point is space $r_i$ and time point $t_k, k \geqslant 1$.

Indeed, if the long-term simulation data live on a low-dimensional, say $m-$dimensional manifold, then $2m+1$ generic observables suffice to embed the manifold, and then learn *any* function on the manifold in terms of these observables. Here we attempted an experiment with a neural network that uses a *local* parametrization of this manifold, testing if such a local parametrization can be learned (in the spirit of the nonlinear discretization schemes of Brenner et al [43]).

**Fig. 8:**
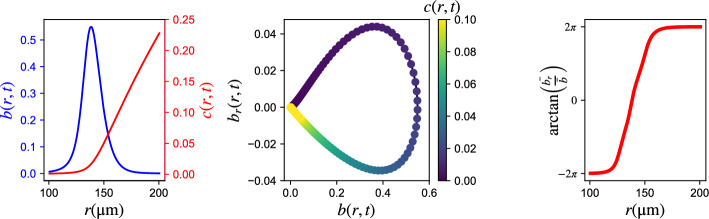
Transformation on the inputs: (left) representative profiles of both fields *b*(*r*, *t*), *c*(*r*, *t*), (middle) visualization of the singularity, (right) transformed variable

There is, however, one particular technical difficulty: because the long-term dynamics of our problem appear in the form of travelling waves, both the front and the back of the wave appear practically *flat* – the spatial derivatives are all approximately zero, and a simple neural network cannot easily distinguish, from local data, if we find ourselves in the flat part *in front* of the wave or *behind* the wave. We therefore constructed an additional local observable, capable of discriminating between flat profiles “before” and flat profiles “after” the wave. Indeed, when the data represent the spatiotemporal evolution of a traveling wave (as in our training/testing data set), we expect a **singularity close to** $\mathbf {b(r,t)=0}$. Clearly, however, the *c* field is dramatically different on the two “flat bacteria” sides (see the left panel of Fig. 8). When learning such a function **locally**, to circumvent this singularity, we proposed using a transformation of two of the inputs: $(b,\nabla b \cdot \hat r) \to \left( {b,\arctan (\frac{{\overline {(\nabla b \cdot \hat r)} }}{{\bar b}}} \right)$, where the bar symbol denotes an affine transformation of the respective entire feature vector to the interval $[-1,1]$. This transformation brings points at different sides of the aforementioned singularity at different ends of a line, exploiting their difference in sign (see Fig. 8).

Then, the Neural Network was trained to learn the estimator (nonlinear observer) of the chemonutrient field as:

8b $$\begin{aligned} c({r_i},{t_k}) = & {C_{NN}}(b({r_i},{t_k}),\arctan \left( {\frac{{\overline {(\nabla b \cdot \hat r)({r_i},{t_k})} }}{{\overline {b({r_i},{t_k})} }}} \right), \\ & (\Delta b)({r_i},{t_k}),b({r_i},{t_{k - 1}}),\,\,(\nabla b \cdot \hat r)({r_i},{t_{k - 1}}),(\Delta b)({r_i},{t_{k - 1}})), \\ \end{aligned}$$

for any discretization point is space $r_i$ and time point $t_k, k \geqslant 1$.

**Fig. 9:**
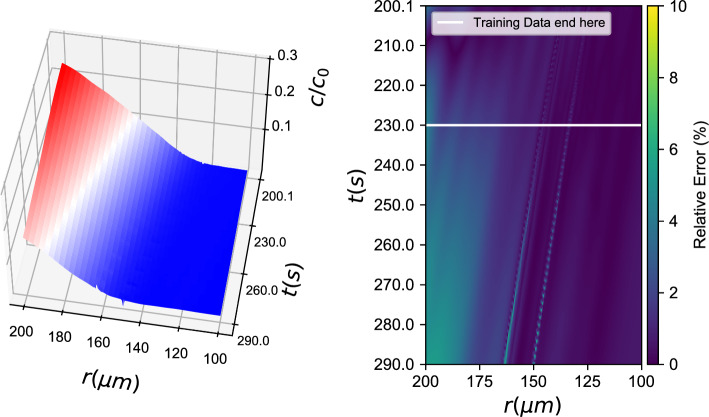
Learning the c-field with a Neural Network: (left) Field prediction and (right) % relative error

As it can be seen from Fig. 9 through the model in Eq. 8b it was possible to provide reasonable predictions for the chemonutrient field.

### Black box model - experimental data

The chemotactic motion of bacteria can also be studied through laboratory experiments. As shown in [1], chemotactic motion can be tracked using confocal fluorescence microscopy of *E. coli* populations; thus, we used the data from these prior experiments here. As detailed further in [1], we 3D-printed a long, cylindrical inoculum of densely-packed cells within a transparent, biocompatible, 3D porous medium comprised of a packing of hydrogel particles swollen in a defined rich liquid medium. Because the individual particles were highly swollen, their internal mesh size was large enough to permit free diffusion of oxygen and nutrient (primarily *L*-serine, which also acts as a chemonutrient), but small enough to prevent bacteria from penetrating into the individual particles; instead, the cells swam through the interstitial pores between hydrogel particles.

We hypothesized that the spatiotemporal behavior of cell density observed in the experiments results from a PDE similar to the one used in simulations (Eq. 10). However, as the authors of [1] examine, several corrections are necessary in order to account for cell crowding, nutrient depletion and cell growth. In addition, spatiotempotal observations of the nutrient concentrations were not experimentally feasible to measure. Having no measurements of the spatiotemporal evolution of the chemonutrient, we turned to the methodology described earlier for data-driven models with partial information.

Interpolation in time allowed for well-approximated time derivatives as we could choose data along *t* as dense as necessary. In fact, it was possible to estimate second order in time derivatives, which could be used to learn a second order in time continuous-time PDE in lieu of a delay model [39], such as that used in Eq. 5:

9a $${b_{tt}} = f_{NN}^{\exp }(b,\nabla b \cdot \hat r,\Delta b,{b_t})$$

This was treated as two coupled first-order PDEs, using the intermediate “velocity” field *v*(*r*, *t*):

9b $${v_t} = f_{NN}^{\exp }(b,\nabla b \cdot \widehat r,\Delta b,u), \hspace{0.2cm}{\text{ }}{b_t} = v{\text{ }}$$

Given the nutrient-starved/hypoxic conditions at $r \approx 0$ (see Materials and methods), our training data were selected away from the origin. We prescribed bilateral boundary corridors to provide data-informed boundary conditions when integrating the learned PDE.

**Fig. 10:**
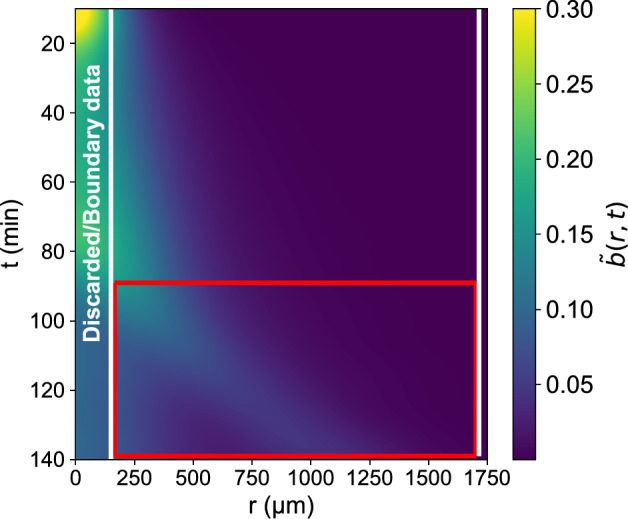
Segmentation of the pre-processed data into: boundary corridors/ discarded data, training subset (the complement), subset chosen for reproduction (red rectangle)

**Fig. 11:**
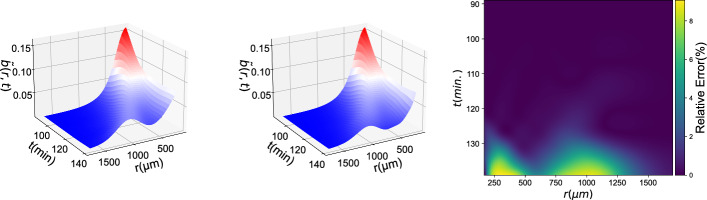
Reproduction of experimental observations using a Data-driven Neural Network for the traveling wave regime: (left) Ground Truth, (middle) Neural-Network PDE integration results, (right) % Relative Error

The model was validated by integration in the spatiotemporal domain of the formation and propagation of the traveling wave (shown in red in Fig. 10). Integration results can be seen in Fig. 11.

## Discussion

Several observations arise from comparing results of different models or methodologies presented in Models and Results.

- For the model in Eq. 3, where both PDEs are data-driven it is obvious that the *c*(*r*, *t*) PDE is easier to learn and predict than the *b*(*r*, *t*) PDE, both for the ANN and the GPR (see, for example Figs. 4, 5 or the Supplementary Information). This can be understood in terms of complexity of these two target functions: The $b_t$ expression is highly nonlinear (owing mostly to the logarithmic chemotactic term) and complex, as it depends on most of the input features. On the contrary the $c_t$ expression only depends on a handful of inputs and is less complicated.
- When learning both PDE right-hand sides (model in Eq. 3), it is straightforward to employ an ANN for multiple output prediction. However, multiple output GP (or cokriging) is non-trivial [44] and therefore, multiple single-output GPs are preferred instead.
- When comparing the ANN methodology with the GPR (for example in Figs. 4, 5 or in the Supplementary Information), for the same model, it can be seen that ANNs tend to produce more accurate predictions than GPR. This may be attributed to the ANN’s versatility and efficiency in capturing the nonlinearities and complexities of any target function. We should also mention that, due to memory constraints, GPR was trained only with an appropriately chosen subset of the training data. This could cause the loss of accuracy in long term predictions. However, it is important to note, that the error in GPR is always *smooth*, owing to the smoothness of the Gaussian kernel (see Supplementary Information).
- Comparing the predictions for $b_t$ from models in Eqs. 3 and 4 it can be seen that when only *one* of the PDEs is data-driven, the predictions are more accurate. This may be rationalized in terms of error accumulation during integration: when both PDEs are data-driven, both of the PDEs contribute to prediction error which will propagate along the integration trajectory.
- The models with partial information were trained with a single (therefore, fixed) delay ($t_k-t_{k-1}$), which imposes important restrictions on how we can advance in time. A natural way to do this, is with a Forward Euler scheme with a timestep equal to the delay used in training, as explicitly shown in Eq. 5.

Observations for models for simulations data are aligned with those for the experimental data: A second order model can indeed capture the dynamics of a data set with partial information. In this case, a deeper Neural Network is required to capture the real-world dynamics from experimental observations. The data-driven PDE manages to capture important characteristics of the traveling wave, such as its speed and amplitude. It also manages to capture the dynamics *on the left* of the traveling wave: the bacteria density remains stationary, as in that region the chemonutrient gradient is negligible. Note that, as discussed in [1], analytical models fail to capture this behavior without non-autonomous correction terms.

## Conclusions

We demonstrated how data-driven methods are able to learn macroscopic chemotactic PDEs from bacterial migration data. The methodology presented was applied either to data sets from fine PDE simulations or from experiments. For an example with agent-based computations see [9]. The same task can be accomplished even when the data at hand are partial, noisy, and/or sparse. It is also possible to learn just one term of a PDE, or a certain PDE out of a set of coupled PDEs. These data-driven models were able to reproduce spatiotemporal profiles used for training and extrapolate further in time. This work showcases that data-driven PDEs are a versatile tool which can be adjusted and implemented to many different problem settings, data sets or learning objectives. It can be especially useful (if not necessary) when the derivation of an analytical PDE is cumbersome, or when there is no capacity for a large number of simulations or experiments. In fact, data-driven PDEs can be used to estimate transients from different initial conditions (IC), for different boundary conditions (BC) or different spatial domain geometries. Apart from that, learning data-driven PDEs is one of the most compact and generalizable ways to learn a system’s behavior from data.

Future work includes relaxing some of the assumptions used here; for example, it is possible to train these models in a *coordinate - free* way [45, 46]. This would result in data-driven PDEs which are valid under coordinate changes (e.g. Cartesian, polar, spherical). That is possible by expressing the PDE in terms of the exterior derivatives. Please refer to the Supplementary Information for a brief outlook on the use of differential operators arising from exterior calculus [47, 48] to create a dictionary of features in which to express learned operators.

In addition, it is also possible to limit the number of independent, relevant inputs in a data-driven way, using dimensionality reduction, automatic relevance determination or other feature importance methods [49, 50]. These future directions aim in more robust and generalizable data-driven PDEs.

## Materials and methods

### PDE simulations

To model chemotactic motion of *E. coli* in heterogeneous porous media, the following extension of the Keller-Segel model [1] was used:

10 $$\begin{aligned}&\frac{\partial {b}}{\partial {t}} = D_b \Delta b -\chi _0\nabla \cdot [b \nabla log F_1(c)] +b \gamma F_2(c) \nonumber \\&\quad \frac{\partial {c}}{\partial {t}} = D_c \Delta c -b \kappa F_2(c) \nonumber \\&\quad \mathbf { J_b}(0,t)\cdot \hat{{\textbf{n}}} = 0, \mathbf { J_b}(R,t)\cdot \hat{{\textbf{n}}} = 0 \nonumber \\&\quad \mathbf {\nabla c}(0,t) \cdot \hat{{\textbf{n}}} = 0, \mathbf {\nabla c}(R,t)\cdot \hat{{\textbf{n}}} = 0, \end{aligned}$$

**Table 3:** Parameters used for the PDE simulation of the extended Keller-Segel model

Parameter	Value	Unit
$D_b$	2.325	$\mathrm {\upmu m^2 /s}$
$\chi _0$	17.9	$\mathrm {\upmu m^2 /s}$
$c_-$	1	$\mathrm {\upmu M}$
$c_+$	30	$\mathrm {\upmu M}$
$c_{char}$	1	$\mathrm {\upmu M}$
$\gamma$	0	$\mathrm {\upmu M/s/ \upmu m^3}$
$D_c$	800	$\mathrm {\upmu m^2 /s}$
$\kappa$	3000	$\mathrm {\upmu M/s/ \upmu m^3}$
$b_0$	0.95	$1/ \mathrm {\upmu m^3}$
$\sigma$	42.62	$\mathrm {\upmu M}$
$c_0$	10	$\textrm{m M}$
*R*	17.5	$\textrm{m m}$

where in radial coordinates *b*(*r*, *t*) is the bacterial density, *c*(*r*, *t*) is the chemonutrient concentration, $D_b$ is the bacterial diffusion coefficient, $D_c$ is the chemonutrient diffusion coefficient, *R* (in the boundary conditions) is the overall domain radius, $F_1(c)=\frac{1+c/c_-}{1+c/c_+}$ and $F_2(c)= \frac{c}{c+c_{char}}$, $\mathbf {J_b}$ is the bacterial density flux ($\mathbf {J_b} = D_b \nabla b - \chi _0 b \nabla log F_1(c))$ and $\hat{{\textbf{n}}}$ is the normal vector at the domain boundaries. Note that in this case, no flux boundary conditions imply zero gradients for both fields at the circular/cylindrical boundary. Experiments can be performed to individually estimate the parameters $D_b, \chi _0, c_-, c_+, D_c, c_{char}, \gamma$, as in [1] (also see Table 3).

**Fig. 12:**
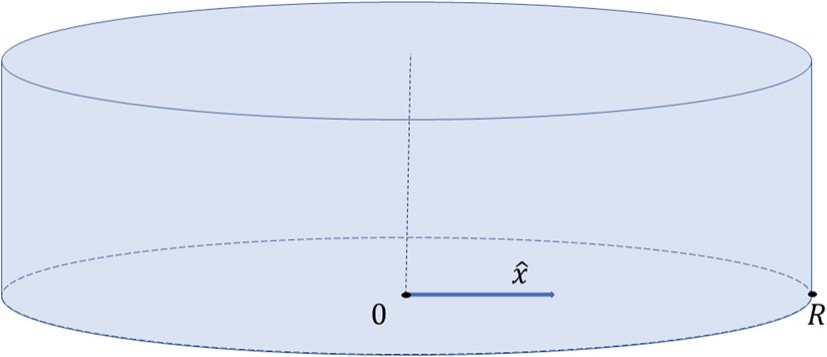
Schematic representation of the computational domain

Numerical simulations of Eq. 10, to provide training data for our data-driven identification approach, were performed using COMSOL Multiphysics 5.5 [51], for the spatiotemporal domain $(t,r) \in [0, 300] \times [0, 1000]$, with initial conditions $b(r, 0) = b_0 e^{-r^2 /2\sigma ^2}, c(r, 0) =c_0$. Note that for all learning cases, the training data are (a suitable subset) in $(t,r) \in [200, 230] \times [0, 250]$ (therefore, $m\cdot n \le 15,801$ in $\mathbb {R}^2$) and the model is validated by simulation in $(t,r) \in [200, 290] \times [0, 250]$. with spatial resolution $dr= 0.5 \mu m$ with the results reported every $\delta t =0.1s$ (relative tolerance set at $10^{-7}$). Integration was performed using Finite Element Method and a MUMPS solver [51]. The parameters of Eq. 10 used in the simulation can be found in Table 3, while a schematic of the computational domain can be found in Fig. 12. Sample code can be found at https://github.com/YorgosPs/Bacterial_chemotaxis.

### Parameter estimation

An artificial input dataset $(r, b,c,{\textbf{p}}$) of size $10^4$ was generated, by perturbing the values obtained from numerical simulations. Then the chemotactic term was evaluated across this dataset. The Neural Network in Eq. 8 was trained with similar hyperparameters as the original gray box model, while the optimization problem in Eq. 9 was solved with a commercial BFGS algorithm.

### Description of the experiments

The cells constitutively express fluorescent protein in their cytoplasms, enabling us to track their motion as they expanded radially outward from the initial cylindrical inoculum in 3D. The fluorescence measurements were collected with spatial resolution $dr =2.48 ~\mathrm {\mu m}$ and temporal resolution $dt =10~\textrm{min}$, and were then azimuthally averaged, only considering signal from the transverse, not the vertical, direction. The fluorescence signal thereby determined is directly proportional to the density of metabolically-active bacteria, and is denoted as $\tilde{b}(r,t)$; cells are left behind in the wake of the moving chemotactic front, but become immobilized and lose fluorescence as they run out of oxygen and nutrients. More information can be found in [1].

### Artificial neural networks

Artificial Neural Networks (ANNs) are a family of functions constructed by composing many affine and nonlinear elementary functions (activation functions). In (feed-forward) neural networks, a universal approximation theorem [52] guarantees that for a single hidden layer with (sufficient) finite number of neurons, an approximation $\tilde{y}$ of the target function, *y* can be found. Here, approximation implies that the target and learned functions are sufficiently close in an appropriately chosen norm: for all $\epsilon >0$ there exists an ANN predicting $\tilde{y}({\textbf{x}})$, where $: |y({\textbf{x}}) -\tilde{y}({\textbf{x}})| <\epsilon$ for all ${\textbf{x}} \in A$ and some $A \subseteq \mathbb {R}^d$. The approximate form of the target function obtained through the feedforward neural net can be written as:

11 $$\begin{aligned} \tilde{y}({\textbf{x}}) =\sum _{i=1} ^{N_n} \psi ({\textbf{w}}_i^T {\textbf{x}} + {\textbf{b}}_i), \end{aligned}$$

where $\psi$ is a nonlinear activation function, ${\textbf{w}}_i, {\textbf{b}}_i$ are tunable parameters (weights and biases) and $N_n$ is the number of neurons, which is decided a priori. To find optimal weights and biases, an optimizer is used (employing a backpropagation scheme) to minimize the root-mean-square error cost function:

12 $$\begin{aligned} E_D=\frac{1}{n} \sum _{j=1}^n (y_j -\tilde{y}(x_j))^2, \end{aligned}$$

which typically measures the goodness of the approximation.

### ANN training and integration

As an example, we mention here the training details regarding one of the models, the one in Eq. 3. Training was performed with a feedforward neural network consisting of two hidden layers, each with 18 tanh-activated neurons. An Adams optimizer [53] was used with a MSE loss function. The neural network hyperparameters were empirically tuned: 2048 epochs and 0.02 learning rate. After training, the data-driven PDE was integrated with a commercial BDF integrator, as implemented in Python’s solve_ivp [54], with relative and absolute tolerances at $(10^{-4}, 10^{-7})$. The initial profile for integration was supplied by our simulation data, and the boundary conditions set to no flux. The Jacobian of the data-driven PDE was provided via automatic differentiation.

### Preprocessing and ANN training for experimental data

**Fig. 13:**
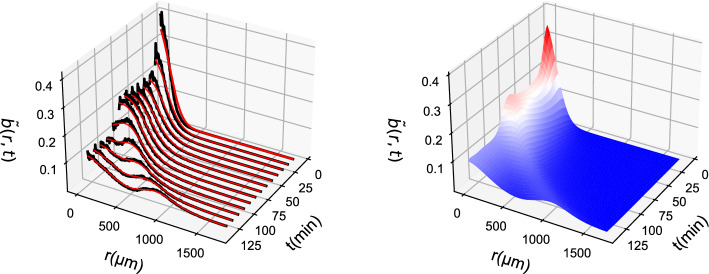
Pre-processing of experimental measurements: (left) Smoothing in space, (right) Interpolation in time

The training profiles were selected appropriately so that the traveling wave is not too close to the spatial boundaries, and the cylindrical coordinate system remains valid. Profiles were smoothed in space using a local Savitzky-Golay filter and globally using Gaussian Smoothing [55, 56]. The resulting smooth profiles were interpolated in time using Gaussian Radial Basis Functions [57] (see Fig. 13).

The learning algorithm consists of a Deep feed-forward Neural Network with 3 hidden layers and 90 neurons per layer. When integrating the data-driven model, SVD filtering was used: $u_t$ is projected to a lower-dimensional space, defined by the dominant singular vectors of the the $u_t(r,t)$ data used in training [58]. This is a procedure analogous to adding hyperviscosity in hydrodynamic models [59]. Here, the eight first singular vectors were used, containing $>99\%$ of the variance.

### Gaussian process regression

To learn a function *f* from data we can employ Gaussian Process Regression (GPR). GPR assumes that the target function $f({\textbf{x}}), f:\mathbb {R}^n \rightarrow \mathbb {R}$ is distributed according to a Gaussian process, which can be fully specified by its mean function $m({\textbf{x}})$ and covariance function $k({\textbf{x}}, \mathbf {x'})$ [49]:

13 $$f({\mathbf{x}})\sim {\mathcal{N}}(m({\mathbf{x}}),k({\mathbf{x}},{\prime })).$$

This can be understood as setting a Gaussian prior distribution over the space of functions. The mean is usually set to zero by centering the data during preprocessing. The covariance function $k({\textbf{x}}, \mathbf {x'})$ is commonly formulated by a Euclidean-distance kernel function in the input space [17]. Here, we use the Matèrn32 kernel with a constant:

14 $$\begin{aligned} k(\mathbf {x_i}, \mathbf {x_j})&= c\left( 1+ \sqrt{3} d(\mathbf {x_i}, \mathbf {x_j};{\textbf{l}} )\right) e^{-\left( \sqrt{3}d(\mathbf {x_i}, \mathbf {x_j};{\textbf{l}})\right) }, \end{aligned}$$

15 $$\begin{aligned} d(\mathbf {x_i}, \mathbf {x_j};{\textbf{l}})&= \sqrt{ \sum _{k=1} ^n \frac{({x_i}_k -{x_j}_k)^2}{l_k }} \end{aligned}$$

where ${\textbf{x}}_i, {\textbf{x}}_j$ are any two feature vectors, *c* is a scalar, ${\textbf{l}}$ is a vector with number of entries equal to the dimension of the input space. *c* and ${\textbf{l}}$ are the hyperparameters to be optimized (here, collectively denoted $\varvec{\theta }$).

Here, we consider the case of noisy observations: $y =f({\textbf{x}}) + \epsilon$, where $\epsilon \sim {\mathcal {N}}(0, \sigma ^2)$ is i.i.d. Gaussian additive noise with known variance. Given a dataset $\{({\textbf{x}}_i, y_i) | i = 1,..., n\}$ the optimal hyperparameter vector $\mathbf {\theta ^*}$ is the maximum likelihood estimator:

16 $$\begin{aligned} \mathbf {\theta ^*}= \arg \min _{\mathbf {\theta }}{\{-log p({\textbf{y}} | {\textbf{x}}, \mathbf {\theta )}}\} \end{aligned}$$

This estimator defines the posterior Gaussian Process given $( {\textbf{x}},{\textbf{y}})$. To find the Gaussian distribution of the function values at test data points, we represent the multivariate Gaussian distribution with the covariance matrix constructed by Eq. 14 as:

17 $$\begin{aligned} \left[ \begin{array}{l} {\textbf{y}} \\ \mathbf {y^*} \end{array} \right] \sim {\mathcal {N}} \left( {\textbf{0}}, \left[ \begin{array}{cc} {\textbf{K}}+ \sigma ^2 {\textbf{I}} & {\textbf{K}}_* \\ {\textbf{K}}^T_* & {\textbf{K}}_{**} \end{array} \right] \right) , \end{aligned}$$

where $${\textbf{y}}^*$$ is a predictive distribution for test data $${\textbf{x}}^*$$, $${\textbf{K}}_*$$ represents a covariance matrix between training and test data while $${\textbf{K}}_{**}$$ represents a covariance matrix between test data. Finally, we represent a Gaussian distribution for the target function at the test points in terms of a predictive mean and its variance, through conditioning a multivariate Gaussian distribution as:

18 $$\begin{aligned} \bar{{\textbf{y}}}^*&= {\textbf{K}}_*({\textbf{K}}+\sigma ^2 {\textbf{I}})^{-1} {\textbf{y}} \end{aligned}$$

19 $$\begin{aligned} {\textbf{K}}({\textbf{y}}^*)&= {\textbf{K}}_{**}- {\textbf{K}}^T_*({\textbf{K}}+\sigma ^2 {\textbf{I}})^{-1} {\textbf{K}}_*, \end{aligned}$$

and we assign the predictive mean ($\bar{{\textbf{y}}}^*$) as the estimated target function for the corresponding data point.

## Supplementary Information

Supplementary Material 1.

## Data Availability

The datasets used and/or analysed during the current study are available at https://github.com/YorgosPs/Bacterial_chemotaxis.

## Abbreviations

PDE: Partial differential equation
(A)NN: (Artificial) neural network
GPR: Gaussian process regression

## References

[1] Bhattacharjee T, Amchin DB, Ott JA, Kratz F, Datta SS. Chemotactic migration of bacteria in porous media. Biophys J. 2021;120(16):3483–97. 10.1016/j.bpj.2021.05.012. 34022238 10.1016/j.bpj.2021.05.012 PMC8391059

[2] Tindall MJ, Maini PK, Porter SL, Armitage JP. Overview of mathematical approaches used to model bacterial chemotaxis ii: Bacterial populations. Bull Math Biol. 2008;70(6):1570–607. 10.1007/s11538-008-9322-5. 18642047 10.1007/s11538-008-9322-5

[3] Sourjik V, Berg HC. Receptor sensitivity in bacterial chemotaxis. Proceed Nat Acad Sci. 2002;99(1):123–7. 10.1073/pnas.011589998. 10.1073/pnas.011589998 PMC117525 11742065

[4] Tu Y. Quantitative modeling of bacterial chemotaxis: signal amplification and accurate adaptation. Annu Rev Biophys. 2013;42(1):337–59. 10.1146/annurev-biophys-083012-130358. (**PMID: 23451887**). 23451887 10.1146/annurev-biophys-083012-130358 PMC3737589

[5] Colin R, Sourjik V. Emergent properties of bacterial chemotaxis pathway. Curr Opin Microbiol. 2017;39:24–33. 10.1016/j.mib.2017.07.004. 28822274 10.1016/j.mib.2017.07.004

[6] Setayeshgar S, Gear CW, Othmer HG, Kevrekidis IG. Application of coarse integration to bacterial chemotaxis. Multiscale Model Simul. 2005;4(1):307–27. 10.1137/030600874.

[7] Siettos C. Coarse-grained computational stability analysis and acceleration of the collective dynamics of a monte carlo simulation of bacterial locomotion. Appl Math Comput. 2014;232:836–47. 10.1016/j.amc.2014.01.151.

[8] Othmer HG, Hillen T. The diffusion limit of transport equations ii: Chemotaxis equations. SIAM J Appl Math. 2002;62(4):1222–50. 10.1137/S0036139900382772.

[9] Lee S, Psarellis YM, Siettos CI, Kevrekidis IG. Learning black- and gray-box chemotactic PDEs/closures from agent based Monte Carlo simulation data. 2022;arXiv. 10.48550/ARXIV.2205.13545. arXiv: https://arxiv.org/abs/2205.13545 10.1007/s00285-023-01946-0 37341784

[10] Narla AV, Cremer J, Hwa T. A traveling-wave solution for bacterial chemotaxis with growth. Proc Natl Acad Sci. 2021;118(48):2105138118. 10.1073/pnas.2105138118. 10.1073/pnas.2105138118 PMC8640786 34819366

[11] Keller EF, Segel LA. Model for chemotaxis. J Theor Biol. 1971;30(2):225–34. 10.1016/0022-5193(71)90050-6. 4926701 10.1016/0022-5193(71)90050-6

[12] Mesibov R, Ordal GW, Adler J. The range of attractant concentrations for bacterial chemotaxis and the threshold and size of response over this range: weber law and related phenomena. J Gen Physiol. 1973;62(2):203–23. 10.1085/jgp.62.2.203. 4578974 10.1085/jgp.62.2.203 PMC2226111

[13] Tu Y, Shimizu TS, Berg HC. Modeling the chemotactic response of Escherichia coli to time-varying stimuli. Proc Natl Acad Sci. 2008;105(39):14855–60. 10.1073/pnas.0807569105. 18812513 10.1073/pnas.0807569105 PMC2551628

[14] Fu X, Kato S, Long J, Mattingly HH, He C, Vural DC, Zucker SW, Emonet T. Spatial self-organization resolves conflicts between individuality and collective migration. Nat Commun. 2018;9(1):2177. 10.1038/s41467-018-04539-4. 29872053 10.1038/s41467-018-04539-4 PMC5988668

[15] Cremer J, Honda T, Tang Y, Wong-Ng J, Vergassola M, Hwa T. Chemotaxis as a navigation strategy to boost range expansion. Nature. 2019;575(7784):658–63. 10.1038/s41586-019-1733-y. 31695195 10.1038/s41586-019-1733-y PMC6883170

[16] Erban R, Othmer HG. From individual to collective behavior in bacterial chemotaxis. SIAM J Appl Math. 2004;65(2):361–91.

[17] Lee S, Kooshkbaghi M, Spiliotis K, Siettos CI, Kevrekidis IG. Coarse-scale PDES from fine-scale observations via machine learning. Chaos: Interdiscip J Nonlinear Sci. 2020;30(1):013141. 10.1063/1.5126869 PMC7043837 32013472

[18] Phan TV, Mattingly HH, Vo L, Marvin JS, Looger LL, Emonet T. Direct measurement of dynamic attractant gradients reveals breakdown of the patlak-keller-segel chemotaxis model. 2023; bioRxiv. 10.1101/2023.06.01.543315. https://www.biorxiv.org/content/early/2023/06/05/2023.06.01.543315.full.pdf 10.1073/pnas.2309251121 PMC10801886 38194458

[19] Rico-Martinez R, Anderson JS, Kevrekidis IG. Continuous-time Nonlinear Signal Processing: A Neural Network Based Approach for Gray Box Identification. 1994; pp. 596–605. https://www.scopus.com/inward/record.uri?eid=2-s2.0-0028723099&partnerID=40 &md5=88de0ca8a967a54274c853e11a84d03f

[20] González-Garcí­a R, Rico-Martí­nez R, Kevrekidis IG. Identification of distributed parameter systems: A neural net based approach. Computers & Chemical Engineering. 1998;**22**, 965–68. 10.1016/S0098-1354(98)00191-4.

[21] Galaris E, Fabiani G, Gallos I, Kevrekidis I, Siettos C. Numerical bifurcation analysis of pdes from lattice boltzmann model simulations: a parsimonious machine learning approach. J Sci Comput. 2022;92(2):34. 10.1007/s10915-022-01883-y.

[22] Raissi M, Perdikaris P, Karniadakis GE. Physics-informed neural networks: A deep learning framework for solving forward and inverse problems involving nonlinear partial differential equations. J Comput Phys. 2019;378:686–707. 10.1016/j.jcp.2018.10.045.

[23] Raissi M, Karniadakis GE. Hidden physics models: machine learning of nonlinear partial differential equations. J Comput Phys. 2018;357:125–41. 10.1016/j.jcp.2017.11.039.

[24] Yang L, Meng X, Karniadakis GE. B-pinns: Bayesian physics-informed neural networks for forward and inverse pde problems with noisy data. J Comput Phys. 2021;425:109913. 10.1016/j.jcp.2020.109913.

[25] Chen RTQ, Rubanova Y, Bettencourt J, Duvenaud D. Neural Ordinary Differential Equations; 2019. arXiv: 1806.07366.

[26] LeCun Y, Bengio Y. Convolutional Networks for Images, Speech, and Time Series. 1998;pp. 255–258. MIT Press, Cambridge, MA, USA.

[27] Rao C, Ren P, Liu Y, Sun H. Discovering Nonlinear PDEs from Scarce Data with Physics-encoded Learning. 2022. arXiv: 2201.12354.

[28] Brunton SL, Proctor JL, Kutz JN. Discovering governing equations from data by sparse identification of nonlinear dynamical systems. Proc Natl Acad Sci. 2016;113(15):3932–7. 10.1073/pnas.1517384113. 27035946 10.1073/pnas.1517384113 PMC4839439

[29] Vlachas PR, Byeon W, Wan ZY, Sapsis TP, Koumoutsakos P. Data-driven forecasting of high-dimensional chaotic systems with long short-term memory networks. Proceed Royal Soc A: Math, Phys Eng Sci. 2018;474(2213):20170844. 10.1098/rspa.2017.0844. 10.1098/rspa.2017.0844 PMC5990702 29887750

[30] Vlachas PR, Arampatzis G, Uhler C, Koumoutsakos P. Multiscale simulations of complex systems by learning their effective dynamics. Nat Mach Intell. 2022;4(4):359–66. 10.1038/s42256-022-00464-w.

[31] Yu J, Lu L, Meng X, Karniadakis GE. Gradient-enhanced physics-informed neural networks for forward and inverse PDE problems. Comput Methods Appl Mech Eng. 2022;393:114823. 10.1016/j.cma.2022.114823.

[32] Li J, Kevrekidis PG, Gear CW, Kevrekidis IG. Deciding the nature of the coarse equation through microscopic simulations: the baby-bathwater scheme. Multiscale Model Simul. 2003;1(3):391–407. 10.1137/S1540345902419161.

[33] Whitney H. Differentiable manifolds. Ann Math. 1936;37(3):645–80.

[34] Takens F. Detecting strange attractors in turbulence. In: Rand D, Young L-S, editors. Dynamical Systems and Turbulence, Warwick 1980. Berlin, Heidelberg: Springer; 1981. p. 366–81.

[35] Stark J, Broomhead DS, Davies ME, Huke J. Takens embedding theorems for forced and stochastic systems. Nonlinear Anal: Theory, Methods Appl. 1997;30(8):5303–14.

[36] Stark J. Delay embeddings for forced systems. IIS deterministic forcing. J Nonlinear Sci. 1999;9(3):255–332. 10.1007/s003329900072.

[37] Stark J, Broomhead DS, Davies ME, Huke J. Delay embeddings for forced systems II. stochastic forcing. J Nonlinear Sci. 2003;13(6):519–77. 10.1007/s00332-003-0534-4.

[38] Sauer T, Yorke JA, Casdagli M. Embedology. J Stat Phys. 1991;65(3):579–616. 10.1007/BF01053745.

[39] Packard NH, Crutchfield JP, Farmer JD, Shaw RS. Geometry from a time series. Phys Rev Lett. 1980;45:712–6. 10.1103/PhysRevLett.45.712.

[40] Aeyels D. Generic observability of differentiable systems. SIAM J Control Optim. 1981;19(5):595–603. 10.1137/0319037.

[41] Altaf MU, Titi ES, Gebrael T, Knio OM, Zhao L, McCabe MF, Hoteit I. Downscaling the 2d bénard convection equations using continuous data assimilation. Comput Geosci. 2017;21(3):393–410. 10.1007/s10596-017-9619-2.

[42] Farhat A, Lunasin E, Titi ES. Continuous data assimilation for a 2d bénard convection system through horizontal velocity measurements alone. J Nonlinear Sci. 2017;27(3):1065–87. 10.1007/s00332-017-9360-y.

[43] Bar-Sinai Y, Hoyer S, Hickey J, Brenner MP. Learning data-driven discretizations for partial differential equations. Proc Natl Acad Sci. 2019;116(31):15344–9. 10.1073/pnas.1814058116. 31311866 10.1073/pnas.1814058116 PMC6681734

[44] Wang B, Chen T. Gaussian process regression with multiple response variables. Chemom Intell Lab Syst. 2015;142:159–65. 10.1016/j.chemolab.2015.01.016.

[45] Luk K, Grosse R. A Coordinate-Free Construction of Scalable Natural Gradient;2020. https://openreview.net/forum?id=H1lBYCEFDB

[46] Weiler M, Forré P, Verlinde E, Welling M. Coordinate Independent Convolutional Networks–Isometry and Gauge Equivariant Convolutions on Riemannian Manifolds. 2021.

[47] Flanders H. Differential Forms with Applications to the Physical Sciences. 2nd ed. New York: Dover Books on Advanced Mathematics. Dover Publications Inc; 1989.

[48] Lee J. Manifolds and Differential Geometry. Graduate Studies in Mathematics, vol. 107. American Mathematical Society, Providence, Rhode Island. 2009. 10.1090/gsm/107. http://www.ams.org/gsm/107 Accessed 2022-04-19.

[49] Rasmussen CE, Williams CKI. Gaussian Processes for Machine Learning. Cambridge, MA: MIT Press; 2006.

[50] Ghorbani A, Abid A, Zou J. Interpretation of neural networks is fragile. Proceed AAAI Conf Artif Intell. 2019;33(01):3681–8. 10.1609/aaai.v33i01.33013681.

[51] Multiphysics C. Introduction to comsol multiphysics®. COMSOL Multiphysics, Burlington, MA, accessed Feb. 1998;**9**, 2018.

[52] Cybenko G. Approximation by superpositions of a sigmoidal function. Math Control Signals Syst. 1989;2(4):303–14.

[53] Kingma DP, Ba J. Adam: A Method for Stochastic Optimization. 2014; arXiv. 10.48550/ARXIV.1412.6980. arXiv: https://arxiv.org/abs/1412.6980

[54] Shampine LF, Reichelt MW. The matlab ode suite. SIAM J Sci Comput. 1997;18(1):1–22. 10.1137/S1064827594276424.

[55] Savitzky A, Golay MJE. Smoothing and differentiation of data by simplified least squares procedures. Anal Chem. 1964;36(8):1627–39. 10.1021/ac60214a047.

[56] Getreuer P. A Survey of Gaussian Convolution Algorithms. Image Process Line. 2013;3:286–310. 10.5201/ipol.2013.87.

[57] Fasshauer GE. Meshfree Approximation Methods with Matlab. World Scientific, Illinois Institute of Technology, USA);2007. 10.1142/6437. https://www.worldscientific.com/doi/pdf/10.1142/6437.

[58] Halko N, Martinsson PG, Tropp JA. Finding structure with randomness: Probabilistic algorithms for constructing approximate matrix decompositions;2010. arXiv: 0909.4061.

[59] Thiem TN, Kemeth FP, Bertalan T, Laing CR, Kevrekidis IG. Global and local reduced models for interacting, heterogeneous agents. Chaos: Interdiscip J Nonlinear Sci. 2021;31(7):073139. 10.1063/5.0055840. 10.1063/5.0055840 34340348

