# Supplementary material for: Data-driven discovery of chemotactic migration of bacteria via coordinate-invariant machine learning
Source: BMC Bioinformatics. 2024 Oct 24;25:337. doi: 10.1186/s12859-024-05929-w (PMC11515320; doi:10.1186/s12859-024-05929-w)
Supplement: Supplementary file 1 — Supplementary Material 1. [file 12859_2024_5929_MOESM1_ESM.pdf]

Supplementary Information: Data-driven Discovery of Chemotactic Migration of Bacteria via Coordinate-invariant Machine Learning

Yorgos M. Psarellis[a], Seungjoon Lee[b], Tapomoy Bhattacharjee[c], Sujit S. Datta[d], Juan M. Bello-Rivas[a], Ioannis G. Kevrekidis[a,e,f,*]

[a] *Department of Chemical and Biomolecular Engineering, Johns Hopkins University*
[b] *Department of Mathematics and Statistics, California State University, Long Beach*
[c] *Andlinger Center for Energy and the Environment, Princeton University*
[d] *Department of Chemical and Biological Engineering, Princeton University*
[e] *Department of Applied Mathematics and Statistics, Johns Hopkins University*
[f] *Department of Medicine, Johns Hopkins University*

1. PDE models not descibed in the text

1.1. Black box learning of both PDEs with GPR (with fields $b(r,t), c(r,t)$ known).

$$\begin{bmatrix} b_t \\ c_t \end{bmatrix} = \begin{bmatrix} f_{GP}(b, \nabla\mathbf{b}\cdot\hat{\mathbf{r}}, \Delta b, c, \nabla\mathbf{c}\cdot\hat{\mathbf{r}}, \Delta c) \\ h_{GP}(b, \nabla\mathbf{b}\cdot\hat{\mathbf{r}}, \Delta b, c, \nabla\mathbf{c}\cdot\hat{\mathbf{r}}, \Delta c) \end{bmatrix} \tag{1}$$

* Corresponding author
Email address: yannisk@jhu.edu (Ioannis G. Kevrekidis)

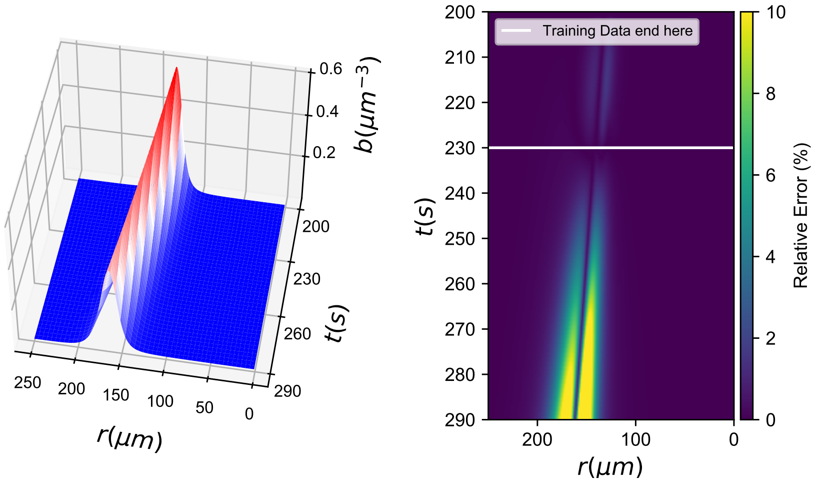

Figure 1: Black box learning of both PDEs with Gaussian Process Regression: (left) Integration results for the **first** data-driven PDE (for $b(r,t)$) and (right) relative error (%). Note that the white horizontal line separates the training dataset from the rest of the validation dataset.

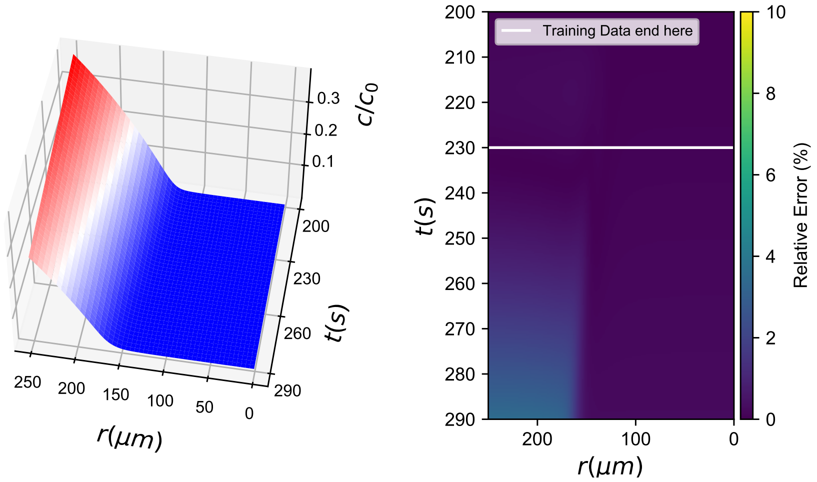

Figure 2: Black box learning of both PDEs with Gaussian Process Regression: (left) Integration results for the **second** data-driven PDE (for $c(r,t)$) and (right) relative error (%).

1.2. Black box learning of b_t - partial information with GPR (with only field $b(r,t)$ known)

$$b(t_{k+1}) = b(t_k) + \Delta t f_{GP}^{partial}(b(t_k), (\nabla \mathbf{b} \cdot \hat{\mathbf{r}})(t_k), (\Delta b)(t_k), b(t_{k-1}), (\nabla \mathbf{b} \cdot \hat{\mathbf{r}})(t_{k-1}), (\Delta b)(t_{k-1})), \tag{2}$$

with $\Delta t = t_{k+1} - t_k$, for any time point $t_k, k \geqslant 1$.

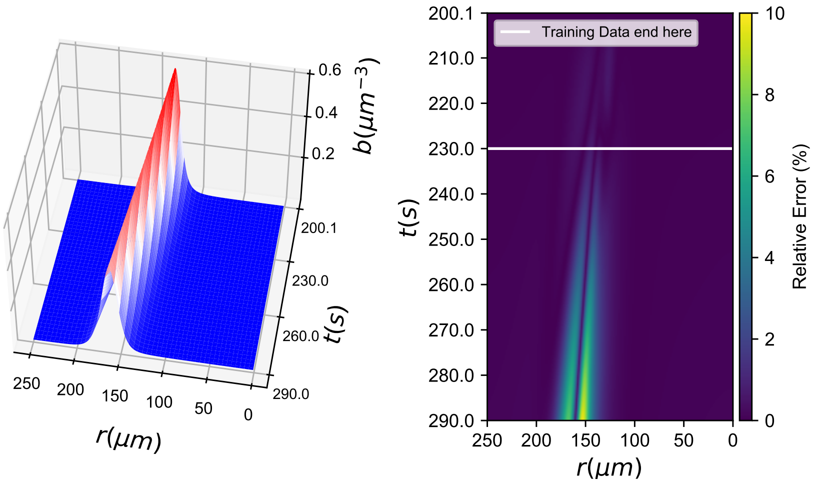

Figure 3: Black box partial-information learning with Gaussian Process Regression: (left) Integration results for the data-driven PDE and (right) % relative error.

1.3. Gray box learning with GPR - c_t known (with fields $b(r,t), c(r,t)$ known).

$$b_t - D_b \Delta b = g_{GP}(b, \nabla \mathbf{b} \cdot \hat{\mathbf{r}}, \Delta b, c, \nabla \mathbf{c} \cdot \hat{\mathbf{r}}, \Delta c) \tag{3}$$

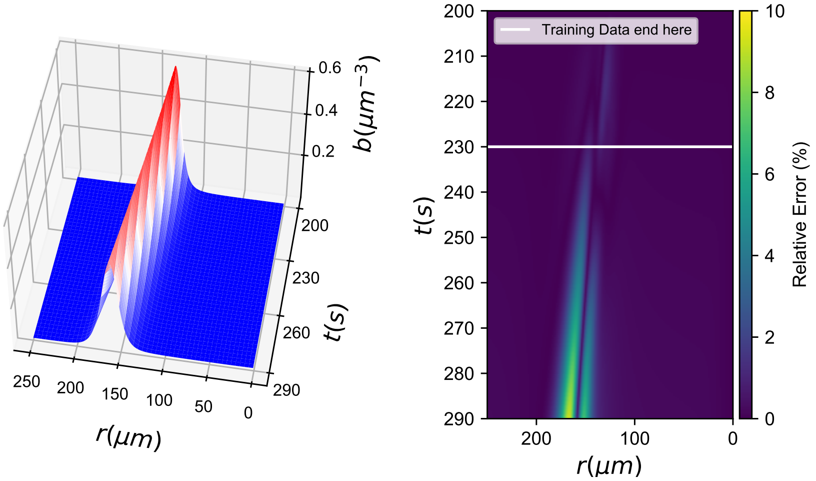

Figure 4: Gray box learning with Gaussian Process Regression: (left) Integration results for the data-driven PDE and (right) % relative error.

1.4. Gray box learning - partial information with GPR (with only field $b(r,t)$ known).

$$b(t_{k+1}) = b(t_k) + \Delta t(D_b \Delta b(t_k) + g_{GP}^{partial}(b(t_k), (\nabla \mathbf{b} \cdot \hat{\mathbf{r}})(t_k), (\Delta b)(t_k), b(t_{k-1}), (\nabla \mathbf{b} \cdot \hat{\mathbf{r}})(t_{k-1}), (\Delta b)(t_{k-1}))), \tag{4}$$

with $\Delta t = t_{k+1} - t_k$, for any time point $t_k, k \geqslant 1$.

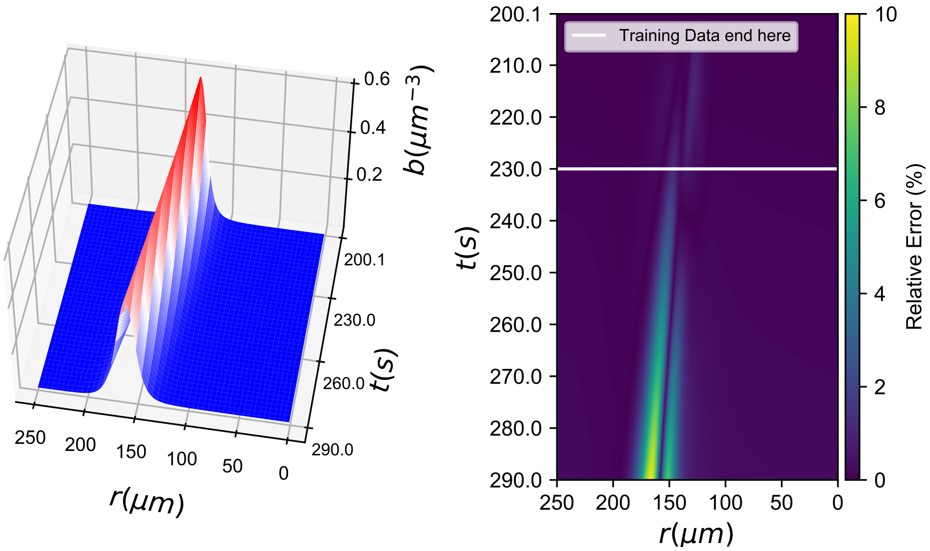

Figure 5: Gray box partial-information learning with Gaussian Process Regression: (left) Integration results for the data-driven PDE and (right) % relative error.

1.5. Gray box learning - partial information b with ANN (with only field $b(r, t)$ known).

$$b(t_{k+1}) = b(t_k) + \Delta t(D_b \Delta b(t_k) + g_{NN}^{partial}(b(t_k), (\nabla \mathbf{b} \cdot \hat{\mathbf{r}})(t_k), (\Delta b)(t_k), \quad b(t_{k-1}), (\nabla \mathbf{b} \cdot \hat{\mathbf{r}})(t_{k-1}), (\Delta b)(t_{k-1}))), \tag{5}$$

with $\Delta t = t_{k+1} - t_k$, for any time point $t_k, k \geqslant 1$.

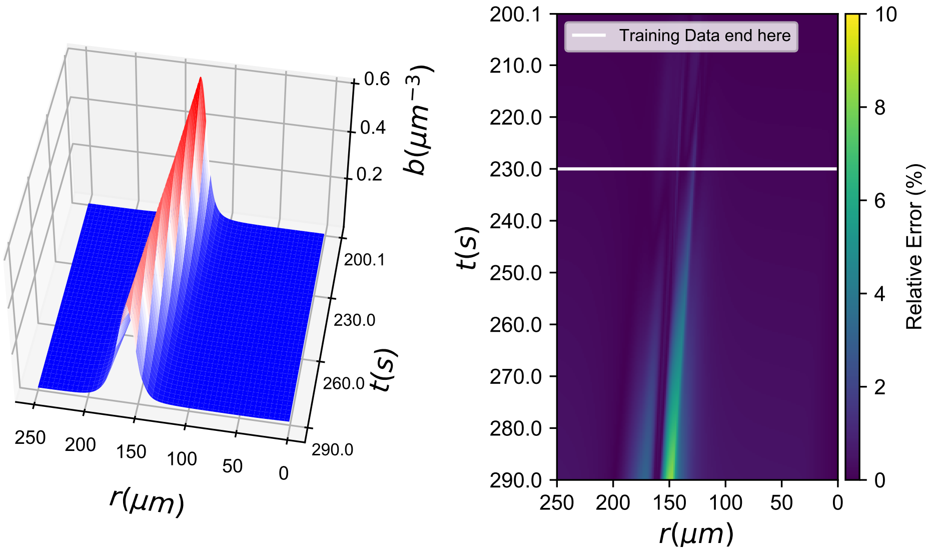

Figure 6: Gray box partial-information learning with a neural network: (left) Integration results for the data-driven PDE and (right) % relative error.

1.6. GPR - Learning c (with only field $b(r,t)$ known).

$$c(r_i,t_k) = C_{GP}\Big(b(r_i,t_k), \arctan\left(\frac{\overline{(\nabla \mathbf{b}\cdot\hat{\mathbf{r}})(r_i,t_k)}}{\overline{b(r_i,t_k)}}\right), (\Delta b)(r_i,t_k), \\ b(r_i,t_{k-1}), (\nabla \mathbf{b}\cdot\hat{\mathbf{r}})(r_i,t_{k-1}), (\Delta b)(r_i,t_{k-1})\Big), \tag{6}$$

for any discretization point is space r_i and time point $t_k, k \geqslant 1$.

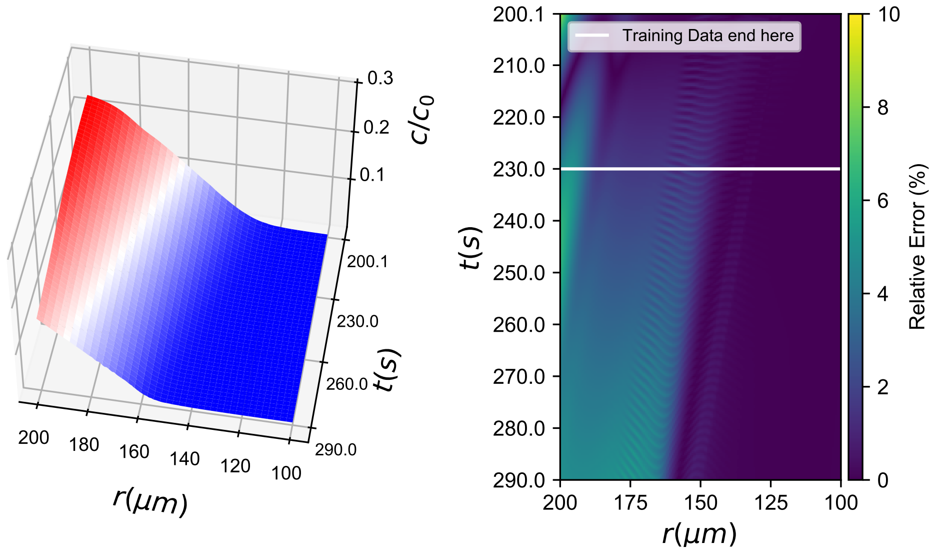

Figure 7: Learning the c-field with Gaussian Process Regression: (left) Field prediction and (right) % relative error.

2. Coordinate-independent operator learning with exterior derivatives

The gradient, curl, divergence, and Laplacian all arise by combining the exterior derivative d, the metric tensor g, the exterior product $\wedge$ and the inner product of differential forms $\langle \cdot, \cdot \rangle$, the Hodge star operator $\star$, as well as the musical isomorphisms $\sharp$ and $\flat$. Consequently, we can extend the problem of learning f in

$$u = f(u, \operatorname{grad} u, \operatorname{div} \operatorname{grad} u, \dots) \tag{7}$$

to the more general setting of learning a function f defined by compositions of the operators mentioned above. For instance, the Navier-Stokes equations [1]

can be written in coordinate-free form as [2]

$$\begin{cases} \dfrac{\partial \omega}{\partial t} = - \star (\omega \wedge \star \mathrm{d}\omega) - \nu \mathrm{d} \star \mathrm{d}\omega + \frac{1}{2}\mathrm{d}\langle \omega, \omega \rangle - \mathrm{d}p \\ \mathrm{d} \star \omega = 0, \end{cases} \tag{8}$$

where $\nu \geq 0$ is the viscosity and p is the pressure.

The use of exterior calculus and exterior differential systems [3] in Physics-informed neural networks is currently growing [4, 5, 6, 7] and a more in-depth study of this framework is an interesting problem for future work.

3. Examples of coordinate-independent learning

In this section , some examples are presented that showcase how the algorithmic framework presented in the main text can generalize in different coordinate systems and/or different domains. In Fig. 8 the neural network in Eq. 4 (main text), that was trained on data in 1D radial coordinates, is able to provide reasonable predictions in 1D Cartesian coordinates (left panel). For comparison, a separate neural Network was also trained on data in 1D Cartesian coordinates and tested on a dataset with 1D radial coordinates (right panel).

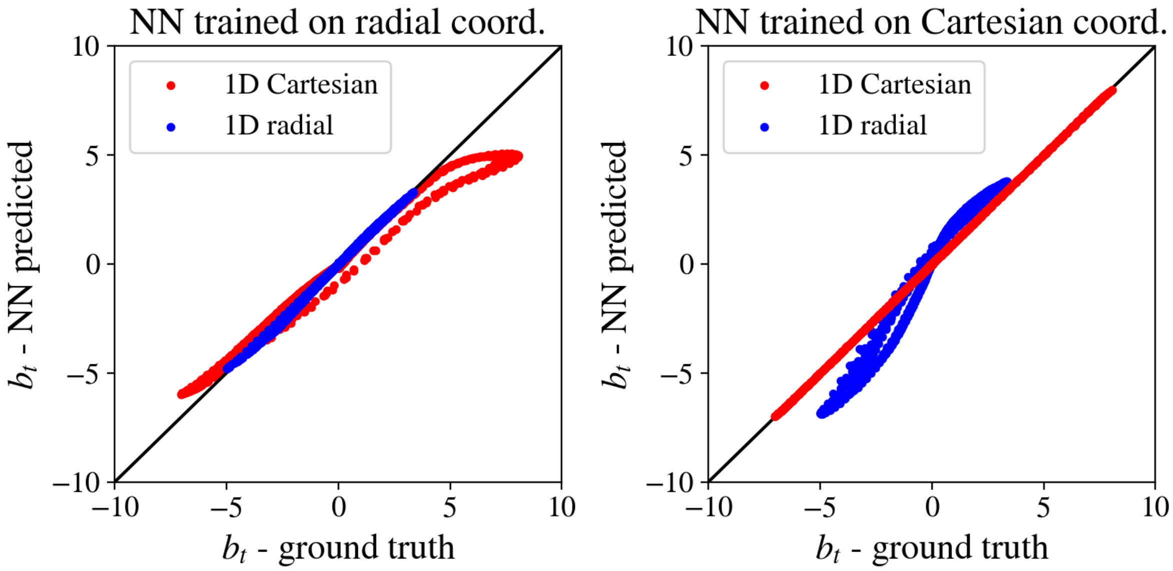

Figure 8: Neural networks trained (and validated) with data from a specific coordinate system can be used to provide reasonable predictions for a different coordinate system: (left) parity plot for a NN trained on radial coordinates simulation data (right) parity plot for a NN trained on Cartesian coordinates simulation data.

Furthermore, in Figs. 9, 10 we show how our approach performs in a more complex scenario, i.e. a nonlinear chemotactic traveling front in 2D (Cartesian coordinates). This trajectory is initialized with uniform nutrient concentration and a narrow, tilted corridor of bacterial density in the middle of a rectangular spatiotemporal domain. After some time, two traveling fronts form, traveling across the large axis of the rectangular domain (here only one of the front is shown). A neural network was trained with the same architecture and input-output features as in Eq. 4 of the main text. Note that all new simulations presented here are based on the same set of PDEs as in the main text (Eqs. 10). In Fig. 9 it is shown how the neural network can accurately predict time derivatives of bacterial density in 2D. In Fig. 10 the same neural network is integrated to reproduce a short 2D trajectory of chemotactic behavior.

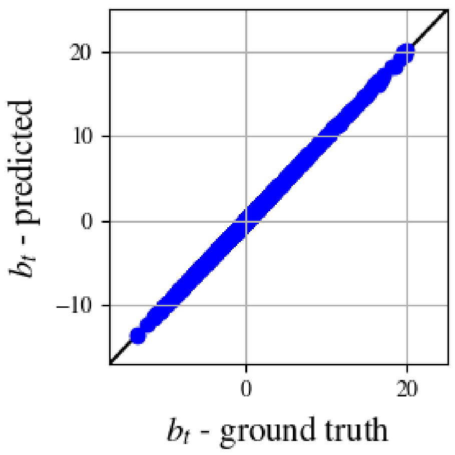

Figure 9: A neural network trained (and validated) with data from an 2D traveling front.

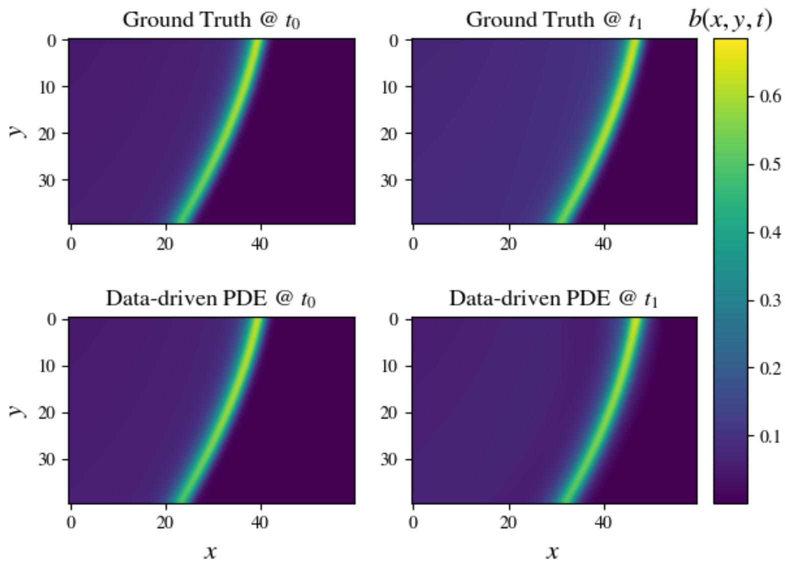

Figure 10: The neural network trained (and validated) with data from an 2D traveling front can be integrated and reproduce more complex chemotactic trajectories in 2D.

References

[1] Gurtin ME. An Introduction to Continuum Mechanics. vol. 158 of Mathematics in science and engineering. New York-London: Academic Press, Inc. [Harcourt Brace Jovanovich, Publishers]; 1981.

[2] Wilson SO. Differential Forms, Fluids, and Finite Models. Proceedings of the American Mathematical Society. 2011 Feb;139(7):2597-604. Available from: http://www.ams.org/jourcgi/jour-getitem?pii=S0002-9939-2011-11003-7.

[3] Bryant RL, Chern SS, Gardner RB, Goldschmidt HL, Griffiths PA. Exterior Differential Systems. vol. 18 of Mathematical Sciences Research Institute Publications. New York, NY: Springer New York; 1991.

[4] Sitzmann V, Martel J, Bergman A, Lindell D, Wetzstein G. Implicit Neural Representations with Periodic Activation Functions. Curran Associates, Inc.; 2020. Available from: https://proceedings.neurips.cc/paper/2020/hash/53c04118df112c13a8c34b38343b9c10-Abstract.html.

[5] Weiler M, Forré P, Verlinde E, Welling M. Coordinate Independent Convolutional Networks – Isometry and Gauge Equivariant Convolutions on Riemannian Manifolds. arXiv:210606020 [cs, stat]. 2021 Jun. ArXiv: 2106.06020. Available from: http://arxiv.org/abs/2106.06020.

[6] Jenner E, Weiler M. Steerable Partial Differential Operators for Equivariant Neural Networks; 2021. Available from: https://openreview.net/forum?id=N9W24a4zU.

[7] Bronstein MM, Bruna J, Cohen T, Veličković P. Geometric Deep Learning: Grids, Groups, Graphs, Geodesics, and Gauges. arXiv:210413478 [cs, stat].

2021 May. ArXiv: 2104.13478. Available from: http://arxiv.org/abs/2104.13478.
